# The role of the (in)accessibility of social media data for infodemic management: a public health perspective on the situation in the European Union in March 2024

**DOI:** 10.3389/fpubh.2024.1378412

**Published:** 2024-04-08

**Authors:** Silvan Wehrli, Christopher Irrgang, Mark Scott, Bert Arnrich, T. Sonia Boender

**Affiliations:** ^1^Centre for Artificial Intelligence in Public Health Research, Robert Koch Institute, Berlin, Germany; ^2^Brown University’s School of Public Health, Brown University, Providence, RI, United States; ^3^Digital Health–Connected Healthcare, Hasso Plattner Institute, University of Potsdam, Potsdam, Germany; ^4^Department of Infectious Diseases, Public Health Service Amsterdam, Amsterdam, Netherlands; ^5^Department of Health Sciences, Faculty of Science, Amsterdam Public Health Research Institute Amsterdam and Amsterdam Institute for Immunology and Infectious Diseases, Vrije Universiteit, Amsterdam, Netherlands; ^6^Risk Communication Unit, Robert Koch Institute, Berlin, Germany

**Keywords:** infodemic, infodemic management, public health, social media, data access, digital services act, misinformation, disinformation

## Abstract

Public health institutions rely on the access to social media data to better understand the dynamics and impact of *infodemics –* an overabundance of information during a disease outbreak, potentially including mis-and disinformation. The scope of the COVID-19 infodemic has led to growing concern in the public health community. The spread of harmful information or information voids may negatively impact public health. In this context, social media are of particular relevance as an integral part of our society, where much information is consumed. In this perspective paper, we discuss the current state of (in)accessibility of social media data of the main platforms in the European Union. The European Union’s relatively new Digital Services Act introduces the obligation for platforms to provide data access to a wide range of researchers, likely including researchers at public health institutions without formal academic affiliation. We examined eight platforms (Facebook, Instagram, LinkedIn, Pinterest, Snapchat, TikTok, X, YouTube) affected by the new legislation in regard to data accessibility. We found that all platforms apart from TikTok offer data access through the Digital Services Act. Potentially, this presents a fundamentally new situation for research, as before the Digital Services Act, few platforms granted data access or only to very selective groups of researchers. The access regime under the Digital Services Act is, however, still evolving. Specifics such as the application procedure for researcher access are still being worked out and results can be expected in spring 2024. The impact of the Digital Services Act on research will therefore only become fully apparent in the future.

## 1 Infodemic management in the current social media landscape

The overabundance of information (true or false) in both the digital and physical world during a disease outbreak is described as an *infodemic* (1). False or misleading information is commonly referred to as misinformation, independent of the intention to spread such information. Disinformation, on the other hand, entails the intention to deceive (2). Social media platforms such as Facebook and Instagram (both Meta) or X (formerly Twitter) play an important role in infodemics due to their widespread use (3), fostering the rapid and global spread of information (4). Obar and Wildman (5) provide the following definition: *social media platforms are internet-based applications, contain mainly user-generated content, allow to create user-specific profiles and to connect with other users on the platform*. Notably, the definition of social media is debatable and has changed over time (6). The here used definition captures platforms that are most relevant in the context of infodemics, i.e., platforms on which any kind of information, opinion or view can be shared and discussed, not restricted to any medium (e.g., text, image, video).

Managing infodemics during disease outbreaks is essential for public health as misinformation can be harmful to the health of individuals, deteriorate the efficacy of public health measures, and disturb social cohesion (7). In addition to the challenges of misinformation, monitoring the online public discourse, concerns, and information voids is essential to facilitate beneficial data-driven public health actions (8). Examples for infodemics have been found in connection with the outbreaks of SARS [e.g., (9)], A(H1N1) [e.g., (10)], measles virus [e.g., (11)], and SARS-CoV-2 (COVID-19).

The velocity and volume of information spread during the COVID-19 pandemic was unprecedented, rendering this infodemic unique in its scope (1). Social media platforms likely played a pivotal and catalyst role in enabling the extent of the COVID-19 infodemic, due to a significantly higher usage during the outbreak and their algorithms designed for content to go viral (12, 13). The severity of the COVID-19 infodemic has led to initiatives of national and international key public health players to build infodemic management capacities: In a joint effort, the WHO has formulated a research agenda, outlining major research areas such as detecting the emergence and spread of an infodemic through social listening tools (14). The Centers for Disease Control and Prevention have presented the COVID-19 State of Vaccine Confidence Insight Reporting System as a first implemented infodemic surveillance system and as prototype for future systems (15). The national public health institute in Germany, the Robert Koch Institute, has developed a framework for using social listening, building the fundament for infodemic management in Germany (16). The Finnish Institute for Health and Welfare and the Africa Infodemic Response Alliance under the hood of the WHO demonstrated similar efforts (17, 18).

These calls have led to manifold efforts in understanding social media narratives: the WHO has developed a taxonomy to rapidly classify online conversations related to COVID-19 (19), mpox (20), and respiratory pathogens (21). The taxonomies have also served as basis for the WHO’s Early AI-supported Response with Social Listening Platform [EARS (22, 23)], which, unfortunately, had to be discontinued from January 2024. Social media data are also used to improve the understanding of social media’s role in infodemics: how COVID-19-related topics spread online [e.g., (24, 25)], the prevalence of misinformation [e.g., (26, 27)], or misinformation interventions [e.g., (28)]. This, in return, helps to improve data-driven social listening tools based on scientific findings and public health needs. To drive public health research and infodemic management forward, public health (research) institutions rely on data access to major social media platforms. However, the access to data from these platforms has been challenging for public health institutions, especially for non-academic organizations [*cf.* (16)].

Recently, X attracted attention with a paradigm shift after restricting access to its data. In the past, it had provided the research community with a relatively generous research access. In March 2023, X switched to a more restrictive policy (29), which had, in advance, led to public criticism from well-known researchers and organizations (30). Others like TikTok (31) have developed new data access regimes but limit access to almost exclusively academic researchers.

At the same time, a European legal framework, the Digital Services Act [DSA, (32)], has come into force on 16 November 2022 (33). Its aim, in part, is to boost transparency and accountability over social media platform operations. These measures include the creation of binding data access regimes to allow researchers to conduct independent research on how these platforms operate.

In the following, we discuss the challenging access to data from social media platforms within the European Union (EU). We provide an overview on data access programs of major social media platforms included in the DSA. We take the perspective of national public health institutes (such as the Robert Koch Institute in Germany) or international public health agencies in the EU (such as the European Center for Disease Prevention and Control) and qualify our findings in terms of usability for such actors. These organizations are key players in public health crisis management and help shape national and international public health measures. Typically, these organizations conduct research to implement measures that are evidence-based and data-driven, and have therefore a need for accessing relevant data for infodemic management, preparedness and response. In a second part, we contextualize the overview with the DSA, serving as a new legal framework for researchers to access data. We first introduce the aim and functioning of the DSA to then relate its use to public health research.

## 2 Accessing data from major social media platforms

To assess the data accessibility of major social media platforms for public health researchers in the EU, we limited our discussion to platforms that fall under the European Commission’s definition of very large online platforms (VLOPs). To qualify as a VLOP, a platform must have at least 45 million average monthly users in the EU (32), corresponding to roughly 10% of the EU’s total population. Obligations for social media platforms introduced in the context of the DSA only apply to VLOPs, which is why we focused on these platforms. At the time of writing (March 2024), 21 platforms^1^ were designated as VLOPs (34, 35). We then selected social media platforms following the definition presented earlier. From this selection, we excluded Wikipedia (because of its primary function as an encyclopedia) as well as Pornhub and XVideos (because of their specific focus on pornographic content). We included the following platforms: Facebook, Instagram, LinkedIn, Pinterest, Snapchat, TikTok, X, and YouTube. Finally, we gathered information on available data access programs for these platforms based on their websites, as of March 2024. We regarded any dedicated offer provided by the platforms to collect, view, or analyze data as a “data access program.” We did not focus on specific accessibility methods (i.e., application programming interface (API) versus web-based dashboards), aiming to evaluate the general availability of data. Of note, programs exclusively built and used for marketing purposes were not considered relevant to public health practice because of their limited scope. Finally, we did not include alternatives to official programs offered by the platforms such as commercial data aggregators.

Table 1 lists the identified data access programs for all platforms and the number of monthly average users that qualify these platforms as VLOPs. All examined platforms offer at least one data access program. LinkedIn, Pinterest, Snapchat, and TikTok offer one program; Instagram, X, and YouTube two programs; and Facebook three programs. Accessibility criteria can greatly vary between platforms and even for programs of the same platform. The “TikTok Research API” is only available to researchers employed by a university, which excludes researchers at non-academic public health institutions. LinkedIn, Pinterest and Snapchat, on the other hand, allow data access through the DSA, which likely includes access for public health institutions (*cf.* next section). Similarly, X and YouTube offer DSA data access. Additionally, X offers a commercial API-based data access (45), which, however, is reported to possibly cost tens of thousands of dollars per month (55). YouTube’s second research program “YouTube Research Program” only accepts applications from university-based researchers similar to the “TikTok Research API” program. As such, data access from X and YouTube is most likely possible for public health researchers through the DSA, as the other options either exclude non-academic researchers (YouTube) or likely cause unaffordable cost for continued data access (X). Out of the two options for Instagram, the “Meta Content Library and API” is likely accessible for public health institutions being a DSA-conform research program. The other option, CrowdTangle, will be discontinued in August 2024 and no longer accepts new applications. These two options are also available for data access from Facebook and a similar conclusion applies. Facebook’s third option, FORT, is limited to selected partners, and has so far focused on elections and democracy as focus research areas, thus excluding the public health domain.

**Table 1:** Data access programs for social media platforms designated as Very Large Online Platforms (VLOPs), March 2024.

Platform	Monthly average users (in million, self-reported)	Data accessibility and purpose
Facebook (Meta)	258 (49)	CrowdTangle (36–38): access to popular public pages (Facebook), groups and verified profile (Facebook, Instagram); only for Facebook partners, Journalists, research NGOs, and non-governmental, non-profit academic institutions; discontinued from August 14, 2024 (application no longer possible) FORT (39): access to selected datasets related to elections & democracy; only for Facebook partners, currently no applications possible DSA research access through the “Meta Content Library and API” (40, 41): access to full public archive of different meta data
Instagram (Meta)	257 (49)	CrowdTangle (see above) DSA research access through the “Meta Content Library and API” (see above)
LinkedIn	45.23 (50)	DSA research access (42): access to public data upon successful application, scope of data unclear
Pinterest	>45 (43)	DSA research access (43): access to public data upon successful application, scope of data unclear
Snapchat	102 (51)	DSA research access (44): access to public data upon successful application, scope of data unclear
TikTok	134 (52)	TikTok Research API (31): access to account and content data; only for non-profit academic institutions in the U.S. or Europe
X (formerly Twitter)	111.4 (53)	X API (45): access scope depends on commercial access tier; available for any institution DSA research access (46): through a form in the developer and agreement policy, scope of data unclear
YouTube	425.2 (54)	YouTube Researcher Program (47): access to the global YouTube video metadata corpus; only for researchers at academic institutions DSA research access (48): access to public data upon successful application, scope of data unclear

Out of the examined 11 access programs, seven are likely accessible for researchers at public health institutions, resulting in potential data access to seven (Facebook, Instagram, LinkedIn, Pinterest, Snapchat, X, and YouTube) out of the eight examined platforms, but only through the DSA. We point out that data access under the DSA is a new possibility, and the specifics of this data access (e.g., what data can be retrieved from platforms) are still under development, as discussed in the next section.

## 3 The DSA and infodemic management

In the following, we shift the discussion to the DSA and how it can be expected to improve the situation for infodemic management. We first introduce the goal and the fundamental building blocks of the DSA, which is defined in EU Regulation 2022/2065 (56).

The DSA targets illegal online activities and disinformation spread via online intermediaries and platforms like marketplaces and social networks, aiming to safeguard European citizens’ digital rights through clear regulatory standards for digital companies (57). This includes a variety of transparency and accountability requirements for social media platforms in the form of regular reports, outside audits and risk assessments. In September 2023, the targeted companies published their initial transparency reports (58), including details such as the number of content moderators (by EU language) or the amount of content removed over a given time period. These reporting duties represent an important building block of the DSA as it allows to assess the platforms adherence to their legal obligations. The legislation includes fines of up to 6 % of a company’s annual global turnover, and the platforms’ mandatory reports can serve as evidence for the European Commission to start formal investigations into violations of the DSA. So far, this has happened for X in December 2023 (59) and TikTok in February 2024 (60).

A second building block consists of Digital Services Coordinators (DSCs). By February 2024, each EU member country had to designate an individual or a local agency to be its DSC, yet not all countries have done this so far (61). DSCs serve as main contact point for both individuals seeking redress and for cross-border issues (61, 62). The European Commission will chair the European Board for Digital Services, in which representatives from each member country will convene regularly to discuss implementation and enforcement issues related to the DSA (63).

A third building block constitutes the platforms’ obligation to give researcher access to platform data from VLOPs to conduct research on systemic risks, defined in DSA Article 34 (1), including the protection of the public health. The requirements for research data access are outlined in Article 40 of the DSA (56, 64). Article 40(8) states that researchers can apply for the status of “vetted researcher” to the DSC, who then acts as an intermediary between vetted researchers and VLOPs. For a successful application, researchers must meet certain conditions: affiliation with a research organization, independence of commercial interests, disclosure of funding, fulfillment of required data security and confidentiality requirements, research in line with the DSA’s purpose, and open-access research. The definition of a “research organization” is set in in Article 2 (1) in EU Directive 2019/70 (65) and includes, apart from universities, not-for-profit research institutes with a primary goal of scientific research. This likely includes national public health institutes as research is typically a main focus of their work [e.g., as described in the Robert Koch Institute’ mission statement (66)]. Applications for vetted researcher access are expected to be possible later in 2024 (64). In addition, Article 40(12) allows data access of publicly available platform data for research that only fulfill a subset of the criteria for the status of vetted research (i.e., who are not affiliated with a research organization and without the requirement of open-access research).

The DSA lacks clarity in some aspects as it is not specified what platform data VLOPs will need to share with vetted researcher, what is considered “publicly available data” and how this differs from data available for vetted researchers, or what organizations will effectively be considered “research organizations.” The DSA research access programs identified (Table 1) all refer to DSA Article 40(12), so it is not fully clear what data can be expected through these channels. Fittingly, we were only able to find detailed information on data availability for the Meta access program (40). Currently, the European Commission prepares an additional regulation intended to clarify these uncertainties (67). The results are planned to be published in spring 2024 and are based on feedback from more than 130 interested parties from a call for evidence, which, overall, outlines (public) data access needs, formats, and application procedure with a need for action (68). What is more, the case of X raises some doubts in regard to how platforms implement data access in reality so far, given the current investigations into X’s potential failure to give researcher data access (59). It is therefore rather doubtful that the DSA has already had a significant influence on current public health research. This conclusion is consistent with impressions from interviews with members from different public health agencies (personal communication, 2022–2023)^2^: Currently, few public health experts have direct access to existing social media listening tools. Instead, the primary dataset used by researchers — specifically related to COVID-19 analysis — is Google Trends, followed by *ad hoc* research via Facebook private groups and previously scrapped X datasets.

## 4 Conclusion

In this work, we summarized the social media data (in)accessibility for public health institutions in the EU, which is required for current investments in infodemic preparedness at these institutions. We examined eight major social media platforms. We find that data access to potentially seven platforms (Facebook, Instagram, LinkedIn, Pinterest, Snapchat, X, YouTube) is accessible for public health institutes, which in all cases relies on access through the DSA (or, in the case of X, possibly through a commercial option). The remaining platform, TikTok, limits access to academic institutions. Yet, it can be expected that TikTok data accessibility will align with the DSA, which it is required to do. Without considering the DSA-mandated data access, platforms would either not allow any data access at all (LinkedIn, Pinterest, Snapchat) or restrict data access to selected groups of researchers such as academics (Facebook, Instagram, YouTube). As such, the DSA is clearly a step in the right direction: The legislation acknowledges that social media platforms can foster harmful societal developments and must be held accountable. The DSA aims to create equal rights and obligations for platforms and researchers alike, which is a clear departure from the pre-DSA era with very heterogenous access possibilities. However, it is still evolving (e.g., the implementation of research access for vetted researchers) and the full potential will only become apparent once the DSA is fully implemented.

While we have focused on data access of major social media platforms in this work, we point out that the success of managing infodemics goes beyond just having data access to these platforms: the preparation for infodemics also relies on infrastructure, social listening tools, and personnel training (69); the collection and processing of data for social listening raises ethical questions, which need to be adequately addressed (70); (fringe) social media not covered by the DSA may still be relevant (71); and generative AI may change the misinformation landscape in the future substantially (72). Importantly, social media data are one piece of information of the health information ecosystem. To manage infodemics and to tackle mis-and disinformation is a complex endeavor that concerns many stakeholders and must therefore be solved together – which the president of the European Commission, Ursula von der Leyen, has also emphasized at the World Economic Forum in 2024 (73). In the context of social media data, we believe that the DSA may serve as a platform for such collaboration, where the needs and rights of all stakeholders can be considered.

## Data availability statement

The original contributions presented in the study are included in the article/supplementary material, further inquiries can be directed to the corresponding author.

## Author contributions

SW: Investigation, Methodology, Writing – original draft, Writing – review & editing. CI: Methodology, Supervision, Writing – original draft, Writing – review & editing. MS: Investigation, Methodology, Resources, Validation, Writing – original draft, Writing – review & editing. BA: Supervision, Validation, Writing – review & editing. TB: Conceptualization, Methodology, Supervision, Validation, Writing – original draft, Writing – review & editing.

## Footnotes

^1^These platforms are (in alphabetical order): Alibaba, AliExpress, Amazon Store, Apple AppStore, Booking.com, Facebook, Google Play, Google Maps, Google Shopping, Instagram, LinkedIn, Pinterest, Pornhub, Snapchat, Stripchat, TikTok, Wikipedia, X, XVideos, YouTube, and Zalando.

^2^Mark Scott has conducted 13 interviews on the subject of the “Use of social media for infodemic management and how that data was accessed” with interviewees from the Centers for Disease Control and Prevention, European Centre for Disease Prevention and Control, European Commission, European Medicines Agency, Maltese Ministry of Health, Robert Koch Institute, United Nations Children’s Fund, University of Belgrad, and the World Health Organization. The interviews were conducted between October 2022 and May 2023 in a semi structured format, encompassed by 12 set questions, and lasted between 1 and 1:30 h.
